# A Multi-Environment Trial Analysis of Frost Susceptibility in Wheat and Barley Under Australian Frost-Prone Field Conditions

**DOI:** 10.3389/fpls.2021.722637

**Published:** 2021-08-19

**Authors:** Ariel Ferrante, Brian R. Cullis, Alison B. Smith, Jason A. Able

**Affiliations:** ^1^School of Agriculture, Food and Wine, Waite Research Institute, The University of Adelaide, Urrbrae, SA, Australia; ^2^Centre for Biometrics and Data Science for Sustainable Primary Industries, National Institute for Applied Statistics Research Australia (NIARSA), School of Mathematics and Applied Statistics, Faculty of Engineering & Information Sciences, University of Wollongong, NSW, Australia

**Keywords:** spring radiation frost, spike fertility, factor analytic selection tool, interaction classes, *Hordeum vulgare L*., *Triticum aestivum*, genotype by environment interactions (GEI)

## Abstract

Low temperatures during the flowering period of cereals can lead to floret sterility, yield reduction, and economic losses in Australian crops. In order to breed for improved frost susceptibility, selection methods are urgently required to identify novel sources of frost tolerant germplasm. However, the presence of genotype by environment interactions (i.e. variety responses to a change in environment) is a major constraint to select the most appropriate varieties in any given target environment. An advanced method of analysis for multi-environment trials that includes factor analytic selection tools to summarize overall performance and stability to a specific trait across the environments could deliver useful information to guide growers and plant breeding programs in providing the most appropriate decision making-strategy. In this study, the updated selection tools approached in this multi-environment trials (MET) analysis have allowed variety comparisons with similar frost susceptibility but which have a different response to changes in the environment or vice versa. This MET analysis included a wide range of sowing dates grown at multiple locations from 2010 to 2019, respectively. These results, as far as we are aware, show for the first-time genotypic differences to frost damage through a MET analysis by phenotyping a vast number of accurate empirical measurements that reached in excess of 557,000 spikes. This has resulted in a substantial number of experimental units (10,317 and 5,563 in wheat and barley, respectively) across a wide range of sowing times grown at multiple locations from 2010 to 2019. Varieties with low frost overall performance (OP) and low frost stability (root mean square deviation -RMSD) were less frost susceptible, with performance more consistent across all environments, while varieties with low OP and high RMSD were adapted to specific environmental conditions.

## Introduction

Wheat and barley, the two most important temperate cereals, are widely grown in the world in part due to their broad ecological adaptation (Slafer and Rawson, 1994; FAO, 2018). In many regions, crops are frequently exposed to abiotic stress conditions such as drought, both low (frost) and high (heat) temperatures that increases in severity and occurrence during the grain filling phase. Such conditions lead to significantly reduced grain yield, which is largely determined by the number of grains per unit area in durum wheat (De Vita et al., 2007; Giunta et al., 2007; Ferrante et al., 2012), barley (Arisnabarreta and Miralles, 2006), or bread wheat (Fischer, 2007; Reynolds et al., 2009; Sadras et al., 2012; Slafer et al., 2014; Ferrante et al., 2017, 2020). These abiotic stress constraints may also ultimately impact grain quality (Telfer et al., 2018).

To mitigate against yield loss and quality downgrades, growers usually sow their crops earlier with the risk of exposing the reproductive organs of the plants to the highest susceptibility of spring radiation frost with temperature values below 0°C (Foley, 1945). These low temperatures coincide with the most sensitive stage shortly before flowering (i.e., the critical period for floret primordia and grain number determination defined in a species-specific developmental window (Sadras et al., 2019). In wheat, the period most frequently starts with the emergence of the penultimate leaf (20–30 days prior to anthesis) and ends - about 7–10 days after anthesis (Fischer, 1985). In two-rowed barley, it has been reported to be between 40 and 10 days before the heading stage (Arisnabarreta and Miralles, 2008) and can result in significant frost damage in plants during late winter (barley) and early spring (wheat; Frederiks et al., 2011, 2012, 2015). In Australia, economic losses due to frost in major regions of the wheat belt are estimated at $360 M AUD each year (Zheng et al., 2015; Crimp et al., 2016; Collins and Chenu, 2021).

In addition, cereal varieties can be grouped into winter, facultative and spring types (Visioni et al., 2013). In winter type cereals, a prolonged period of cold temperature (vernalisation) is required to initiate flowering. This mechanism prevents the plant from flowering during winter when it is most susceptible to cold stress. Most winter wheats also respond strongly to long photoperiods after their vernalisation requirement is met (Slafer, 1996; González et al., 2002). In contrast, photoperiod is the primary factor controlling the initiation of flowering in spring types (Davidson et al., 1985). Both of these mechanisms, vernalisation and photoperiod, are effective ways for the plant to avoid frost damage (Visioni et al., 2013). Winter type wheats have a high level of frost tolerance during the vegetative growth phase (> −12°C) which is controlled by the Frost Resistance-2 locus on linkage group 5. In proximity of this locus are the *Vrn1* and *CBF* genes, both of which are transcription factors. *Vrn1* regulates vernalisation and cold tolerance inducible genes (Fowler et al., 1996; Thomashow et al., 2001), whilst *CBF* regulates cold tolerance inducible genes (Thomashow, 2010). Post-vernalisation, as the plant transitions into the reproductive stage, the effectiveness of these genes to regulate cold tolerance genes is reduced (Fowler et al., 1996; Dhillon et al., 2010; Al-Issawi et al., 2013). In barley, variation in reproductive frost tolerance was reported to be associated with a locus on chromosome 2H (Reinheimer et al., 2004).

However, screening germplasm in the field using natural frost has been extremely challenging and often fails. This inability to successfully screen consistently in the field has largely been due to the intra- and inter-season variability of frost events (Frederiks et al., 2012). Previous studies carried out have used various methods, including: (i) simulating frost conditions in a custom-built frost chamber by allowing out-of-season screening (Marcellos and Single, 1975; Chen et al., 2009); (ii) by using movable frost shelters in south-eastern Queensland; (iii) manipulating plants to manage natural frost in portable containers (Tshewang et al., 2017); (iv) adopting serial sowing with photoperiod extension; (v) or through the artificial photoperiod gradient method (Frederiks et al., 2012). This latter method can link together the genotypes (at the same trial) with a different maturity type, to enable a similar growth stage to be determined during a natural frost event. In addition, there have been new, sophisticated methods to assess abiotic stresses that include non-destructive approaches such as proximal sensors, terahertz imaging and computed tomography (Fitzgerald et al., 2019; Nuttall et al., 2019; Lee et al., 2020; Schmidt et al., 2020). Typically, such technology comes at a greater economic cost, due to the implementation of appropriate infrastructure and equipment, and the ability to analyze and validate different hypotheses.

Therefore, in order to breed for reduced frost susceptibility, selection methods are urgently required to identify novel sources of frost germplasm, and then transfer that reduced susceptibility into elite varieties. However, in doing so the presence of genotype by environment interactions (GEI; i.e. genotype response to a change in environment) is the major constraint to select the most appropriate germplasm to a target environment (Smith and Cullis, 2018; Cocks et al., 2019). An advanced method of analysis for multi-environment trials (MET) that includes Factor Analytic Selection Tools (FAST) to summarize overall performance and stability to a specific trait across environments could deliver useful information to guide growers and plant breeders in providing the most appropriate decision making-strategy (Smith and Cullis, 2018).

The aim of this study was to use a new set of FAST to provide information on the genetic propensity of a range of commercially available and advanced breeding lines of wheat and barley to tolerate frost under field conditions. This information is based on a comprehensive MET data-set which spans experiments and involved a range of sowing dates grown at multiple locations from 2010 to 2019.

## Materials and Methods

### Experimental Design

The description of the experimental design and statistical methods for a subset of the wheat data (i.e., 17 frost expression experiments (FEEs) from 2010 to 2016 growing seasons) we use in this research were published in Cocks et al. (2019). In this study, we expanded the data-set to also include the 2017–2019 growing seasons in wheat and also included barley FEEs from 2012 to 2019 (Table 1). Please see Supplementary Figure 1 which highlights the approach undertaken.

**Table 1:** Experimental details described in the multi-environment trial (MET) data-set including different states, sites, experiments (26 and 24 in wheat and barley, respectively), times of sowing (TOS; resulting in a total of 126 and 108 different growing conditions in wheat and barley), number of varieties assessed and number of tagged frost events.

State	Site	Year	Experiment^d^	Times of sowing^a^			Number of varieties^b^		Tagged frost events^c^	
				(Wheat)	(Barley)		(Wheat)	(Barley)	(Wheat)	(Barley)
New South Wales (NSW)	Narrabri	2012	nsw12	4	5		30	20	17	15
		2013	nsw13	3	2		29	19	3	2
		2014	nsw14	3	1		39	20	3	1
		2015	nsw15	3	3		44	20	4	3
		2016	nsw16	2	1		41	21	2	1
		2017	nsw17	4	4		58	30	5	5
		2018	nsw18	3	2		53	30	4	3
		2019	nsw19	5	5		66	36	9	9
South Australia (SA)	Loxton	2010	sa10	2	0		28	0	2	0
		2011	sa11	2	0		34	0	2	0
		2012	sa12	6	6		62	47	4	4
		2013	sa13	3	2		64	46	4	2
		2014	sa14	2	5		36	48	2	4
		2015	sa15	6	5		89	48	2	2
		2016	sa16	5	5	92	48	4	3
		2017	sa17	6	6		64	36	5	7
		2018	sa18	6	6		70	36	7	7
		2019	sa19	6	6		74	36	7	7
Western Australia (WA)	Merredin Wickepin	2012	wa12	3	2		44	20	3	1
		2013	wa13	6	5		54	25	5	4
		2014	wa14	8	5		70	35	7	3
	Brookton	2015	wa15	8	8		106	36	6	3
	Wickepin	2016	wa16	7	6		94	36	3	3
	Dale	2017	wa17	8	4		71	36	4	2
		2018	wa18	7	7		60	34	7	6
		2019	wa19	8	7		69	36	8	8

a *Time of sowing. Block is the number of TOS (whole blocks) from mid-April to early-July depending on the experiment*.

b *Two replications per variety within each TOS*.

c *Number of tagged frost events. A unique tagged plot with 30–40 main-shoot or primary-tiller spikes at flowering time, matching each frost event (multiple frost events across consecutive days is considered as one event)*.

d *Frost expression experiments (FEEs) at Narrabri (Lat 30.30°S, Long 149.80°E); Loxton (Lat 34.47°S, Long 140.58°E); Merredin (Lat 31.49°S, Long 118.22°E); Wickepin (Lat 32.70°S, Long 117.50°E); Brookton (Lat 32.37°S, Long 117.00°E); Dale (Lat 32.20°S, Long 116.75°E)*.

Treatments consisted of all combinations of time of sowing (TOS) and varieties within each FEE and arranged in whole blocks, with each whole block containing a rectangular array of plots. Each block may not be spatially adjacent and each whole block was allocated to one TOS and plots within each whole block were allocated to varieties, using a near resolvable sub-block design with two sub-blocks per whole block. This allocation of treatments to plots resulted in a nested design that is similar to a split-plot design (Bailey, 2008), except that the TOS was completely aliased with whole blocks and hence in the following whenever TOS is referred to, it is bracketed with whole blocks.

In addition, not all plots in an FEE are sampled for assessing frost sterility (FS) during the course of the experiment and hence the number of TOS (whole blocks), varieties, sub-blocks, varieties and field plots in the final data-set are usually not the same as the number of levels for each of these factors in the original design.

### Statistical Methods

The recent study by Cocks et al. (2019) reported in detail the approach used for the analysis of FEE MET data-sets, fitting a factor analytic linear mixed model (FALMM) after Smith et al. (2001). The terms included in the FALMM address the aims of the FEEs, the experimental design of the FEEs, the longitudinal nature of the FS measurements within a FEE and also accommodated the sparse sampling of plots within FEEs. Additional terms are included in the FALMM to account for extraneous variation, such as counter and tagger. Sources of variation, which are associated with anatomical factors such as stage of development (SOD; booting and flowering in wheat; flag leaf emergence and flowering in barley) and relevant interactions with treatment factors and longitudinal factors were also considered in the FALMM. Structural sparsity and incomplete recording of extraneous factors for some FEEs implies that some terms cannot be fitted for all FEEs and provide details of the screening process which determined the maximal set of terms which can be fitted to each FEE (Cocks et al., 2019). All models were fitted using the ASReml-R package (Butler et al., 2017) in the R statistical computing environment (R Core Team, 2015). The transformation used was the same as that used by Cocks et al. (2019). We used a simplified linear mixed model for the analysis of plant height and spike length. We note that plant height was measured at the plot level while spike length was measured on an individual spike, hence the residual terms for these analyses were either at the plot level or at the spike level. Empirical best linear unbiased predictors for the main effects of genotypes were derived from the fit of these models for the two ancillary traits, plant height and spike length.

### Description of the Data Sets

The twenty-six (wheat) and twenty-four (barley) FEEs were carried out in three different frost-prone regions across Australia, namely: Southern (Sth), Western (Wst) and Northern (Nth) regions. The FEEs in South Australia (SA: 10 wheat and 8 barley), New South Wales (NSW: 8 and 8) and Western Australia (WA: 8 and 8) are referred to as a MET data-sets labeled by state and year (e.g., nsw12, sa15 and wa17; see Table 1).

Combining all FEEs for each crop resulted in two MET data-sets where the environment is the concatenation of the labels for state and year (eg nsw12, sa10, etc). Here we refer to the environment as the “Experiment” for consistency with the terminology of an FEE. Five FEEs had zero genetic variance: sa11, nsw14, sa14 (for wheat); nsw13, wa16 (for barley) and these were excluded from the MET analysis. Times of sowing ranged from 1 to 8 depending on FEE, resulting in 126 and 108 different growing conditions across sites and years in wheat and barley, respectively (Table 1). Across FEEs, the total number of varieties ranged from 28 to 106 in wheat, while in barley this varied between 20 and 48. The selection of varieties or breeding lines used for this analysis that included different sites and growing seasons were those same varieties tested within the National Variety Trial program in Australia, where the new released breeding lines are assessed for frost susceptibility every growing season, depending on the species-specific environment (Supplementary Table 1). Variety names used across sites and years are available online at the decision making platform: FV-Plus Frost Rankings.^1^ The varietal connectivity across years and states is presented in Table 2, where the numbers on the off-diagonal are the numbers of varieties in common between each pair of years or states, and the numbers on the diagonals are the total number of varieties used in that year or state.

**Table 2:** Variety connectivity showing the number of varieties in common between each pair of years and states (on the off-diagonal) for the MET data-set, while the numbers highlighted in bold on the diagonals are the total number of varieties used in that year and state.

Wheat														
*Year*	2010	2011	2012	2013	2014	2015	2016	2017	2018	2019	Site	NSW	SA	WA
*2010*	**28**	28	14	16	14	15	15	9	6	6	NSW	**108**	108	94
*2011*	28	**34**	18	20	18	19	19	13	10	10	SA	108	**194**	124
*2012*	14	18	**64**	64	56	56	55	42	25	24	WA	94	124	**157**
*2013*	16	20	64	**70**	58	60	59	44	27	26				
*2014*	14	18	56	58	**110**	74	69	56	38	37				
*2015*	15	19	56	60	74	**118**	86	66	50	46				
*2016*	15	19	55	59	69	86	**105**	75	56	51				
*2017*	9	13	42	44	56	66	75	**84**	63	58				
*2018*	6	10	25	27	38	50	56	63	**78**	69				
*2019*	6	10	24	26	37	46	51	58	69	**80**				
Barley														
*Year*	2010	2011	2012	2013	2014	2015	2016	2017	2018	2019	Site	NSW	SA	WA
*2010*	–	–	–	–	–	–	–	–	–	–	NSW	**44**	44	44
*2011*	–	–	–	–	–	–	–	–	–	–	SA	44	**65**	51
*2012*	–	–	**48**	47	46	46	38	25	25	25	WA	44	51	**52**
*2013*	–	–	47	**47**	45	45	38	25	25	25				
*2014*	–	–	46	45	**48**	48	38	25	25	25				
*2015*	–	–	46	45	48	**48**	38	25	25	25				
*2016*	–	–	38	38	*38*	38	**49**	33	33	33				
*2017*	–	–	25	25	25	25	33	**36**	34	34				
*2018*	–	–	25	25	25	25	33	34	**36**	36				
*2019*	–	–	25	25	25	25	33	34	36	**36**				

In addition, the number of spikes analyzed by crop across FEEs at different developmental stages following Zadoks scale (Zadoks et al., 1974) and the total values are shown in Table 3.

**Table 3:** Number of spikes analyzed by crop across FEEs at different developmental stages following Zadoks scale (Zadoks et al., 1974).

		Number of spikes analyzed^a^					
		Wheat			Barley		
Site	FEE^b^	Booting	Flowering	Total	Flag leaf	Flowering	Total
Narrabri	nsw12	4,369	4,971	9,340	1,621	4,430	6,051
	nsw13	812	3,520	4,332	51	1702	1753
	nsw14	1881	3,834	5,715	0	1,539	1,539
	nsw15	2090	5,721	7,811	722	2020	2,742
	nsw16	0	3,226	3,226	0	1,070	1,070
	nsw17	1,380	5,304	6,684	0	6,300	6,300
	nsw18	0	5,607	5,607	0	2,850	2,850
	nsw19	0	8,016	8,016	0	5,638	5,638
Loxton	sa10	0	2,689	2,689	0	0	0
	sa11	0	2,775	2,775	0	0	0
	sa12	6,456	6,975	13,431	6,217	32,835	39,052
	sa13	648	8,174	8,822	729	9,698	10,427
	sa14	0	2,714	2,714	3,077	22,691	25,768
	sa15	6,952	20,030	26,982	0	15,071	15,071
	sa16	14,854	22,034	36,888	2,860	17,459	20,319
	sa17	0	13,915	13,915	0	9,925	9,925
	sa18	0	18,976	18,976	0	9,435	9,435
	sa19	0	19,499	19,499	0	11,966	11,966
Merredin	wa12	2,692	454	3,146	0	1,255	1,255
Wickepin	wa13	4,602	4,292	8,894	3,561	1,501	5,062
	wa14	18,191	1,597	19,788	3,104	480	3,584
Brookton	wa15	4,286	30,839	35,125	0	6,769	6,769
Wickepin	wa16	5,635	11,248	16,883	0	5,500	5,500
Dale	wa17	6,686	8,523	15,209	0	4,627	4,627
	wa18	3,419	20,375	23,794	0	12,202	12,202
	wa19	0	20,607	20,607	0	7,408	7,408

a *Tagged wheat spikes at booting (DC49, Zadoks et al., 1974) and flowering (DC65) stages. In barley, spikes tagged at flag leaf (DC39) and flowering (DC49-52, depending on varieties) stages. A unique plot was tagged with 30–40 main-shoot or primary-tiller spikes at flowering time, matching each frost event (or when in the same week there were consecutive frost events occurring, this was considered as one event). Frost assessment included a wide range of flowering windows across varieties and times of sowing resulting in a substantial number of spikes analyzed within each growing condition (i.e. experiment × TOS)*.

b *Frost expression experiment*.

In wheat, the percentage of frost sterility (FS) was calculated as the ratio between aborted grains considering only the two most proximal grain positions (G1 and G2) with respect to the rachis, and the total grains at the same grain positions (G1, G2) within spikelet × 100. In the case of 2-row barley varieties, FS was calculated as a ratio between aborted and total grains of the central fertile spikelets along the spike. These values and grain number per spike are presented in Table 4.

**Table 4:** Transformed measured of means, 25^th^ and 75^th^ percentile of frost sterility. Grain number per spike (means and range) in wheat and barley crops within each FEE.

FEE^c^	Frost sterility^a^						Grain number per spike^b^			
	Wheat			Barley			Wheat		Barley	
	25th *p*	Mean	75th *p*	25th *p*	Mean	75th *p*	Mean	Range^d^	Mean	Range
nsw12	0.105	0.233	0.474	0.033	0.094	0.293	34	12–52	27	10–39
nsw13	0.289	0.850	1.000	0.081	0.182	0.481	38	14–52	32	12–42
nsw14	0.028	0.079	1.000	0.050	0.200	0.520	34	10–52	27	13–37
nsw15	0.031	0.077	0.143	0.000	0.036	0.077	36	18–60	28	11–40
nsw16	0.068	0.125	0.225	0.037	0.100	0.207	38	20–52	29	13–39
nsw17	0.147	0.286	0.500	0.038	0.103	0.200	34	20–56	31	16–44
nsw18	0.269	0.472	0.731	0.107	0.208	0.375	32	16–48	25	13–41
nsw19	0.211	0.421	0.737	0.037	0.103	0.212	36	6–56	29	16–48
sa10	0.036	0.107	0.265	–	–	–	32	14–46	–	–
sa11	0.000	0.000	0.031	–	–	–	32	12–46	–	–
sa12	0.062	0.115	0.222	0.000	0.045	0.105	28	10–46	25	10–50
sa13	0.029	0.062	0.111	0.034	0.071	0.148	30	12–56	25	10–46
sa14	0.077	0.143	0.250	0.037	0.083	0.190	30	16–40	22	10–47
sa15	0.071	0.150	0.300	0.042	0.087	0.158	24	10–40	23	10–49
sa16	0.115	0.214	0.417	0.043	0.120	0.242	28	10–45	25	10–40
sa17	0.071	0.222	0.864	0.042	0.115	0.409	26	10–44	24	11–39
sa18	0.033	0.083	0.167	0.000	0.050	0.100	26	8–56	21	10–40
sa19	0.233	0.567	1.000	0.083	0.240	0.690	32	8–54	24	10–50
wa12	0.000	0.038	0.091	0.000	0.000	0.048	26	12–38	24	10–37
wa13	0.000	0.033	0.071	0.000	0.000	0.040	28	10–44	23	10–36
wa14	0.000	0.036	0.133	0.000	0.000	0.037	30	10–54	24	10–36
wa15	0.000	0.059	0.167	0.000	0.038	0.105	26	10–58	23	10–36
wa16	0.286	0.821	1.000	0.000	0.056	0.200	30	12–52	22	10–45
wa17	0.000	0.038	0.091	0.000	0.000	0.040	28	12–48	24	10–35
wa18	0.067	0.219	0.630	0.000	0.034	0.077	30	14–54	25	10–39
wa19	0.029	0.077	0.233	0.000	0.033	0.071	28	12–50	24	10–39

a *In wheat, calculated as the ratio between aborted grains considering only the two most proximal grain positions (G1 and G2) with respect to the rachis, and total grains at the same grain positions (G1, G2) within the spikelet*.

b *Minimum and maximum grain number per spike considering only G1 and G2 positions with respect to the rachis within the spikelet*.

c *Frost expression experiment*.

d *Minimum and maximum values*.

### Experimental Conditions and Measurements

The experiments were irrigated to maintain rainfall decile 5 during the growing season. Nitrogen fertilizer was applied following the agronomic practices recommended for each region. Disease and insects were controlled by spraying fungicides and insecticides at the doses suggested by their manufacturers.

In wheat, phenology was recorded at booting (DC49; Zadoks et al., 1974) and/or flowering stages (DC65), while in barley at flag leaf (DC39) and/or flowering stages (DC49-52, depending on varieties) across sites and years. In sa17, sa18 and sa19 experiments, crop phenology was recorded weekly using FieldScorer Software^2^ from sowing (DC00) to physiological maturity (DC89). To accurately determine the flowering stage, a random 2–3 main-shoot spikes within the inner part of each plot (avoiding the edge effect) were selected to determine whether anthers within the floret primordia of the central spikelets were pale green to bright yellow in color, following the Waddington scale (Waddington et al., 1983) and illustrated by Ferrante et al. (2013, 2020).

Spring radiation frost was defined when the air temperature fell below 0°C recorded at the canopy level or 2°C from the Stevenson screen (Bureau of Meteorology) on weather stations located closest to the trials (Jeffrey et al., 2001).

Within forty-eight hours following each frost event or when multiple frost events occurred within the same week (considered in this analysis as one), 30–40 main-shoot or at least primary-tiller spikes per plot were tagged and recorded at the flowering stage with different colored tapener tapes using a tapener gun (Table 3). Plant height at the plot level was recorded from the inner rows of those tagged plots. Depending on crops and experiments, there was also tagged plants at flag leaf (barley) and booting stages (Wheat). Within each experiment and TOS, varieties (and their replications) that reached flowering without matching a frost event were not tagged. However, upon the following frost event, only the remaining varieties that reached flowering later (wide flowering window) were tagged within the same TOS. This methodology was repeated in all TOS within the experiment. Each TOS was delayed by approximately 100°C days.

Tagged spikes were left to develop until the soft dough stage (DC83-85), after which they were individually collected, bagged, labeled and frozen to assess FS in the laboratory (Table 4). Individual spike length was also recorded from the bottom to the tip of the each spike (without considering the awns).

### Climatic Data

In-crop air temperature values (°C), accumulated rainfall (mm day^−1^), and in-crop global radiation (MJ m^−2^) were recorded at daily intervals from weather stations located on the paddock or were obtained from patched point data sets^3^ and compared to long-term data sets from 1960 up to the previous growing season for each FEE. Photo-thermal quotient (the ratio between global radiation and the average temperature (Fischer, 1985) and rainfall decile (e.g., the median or decile 5 or 50th percentile corresponds to the midpoint of the ordered (lowest to highest) monthly or yearly precipitation totals) were also calculated (Supplementary Table 2).

## Results

### Weather Conditions

**Figure 1:**
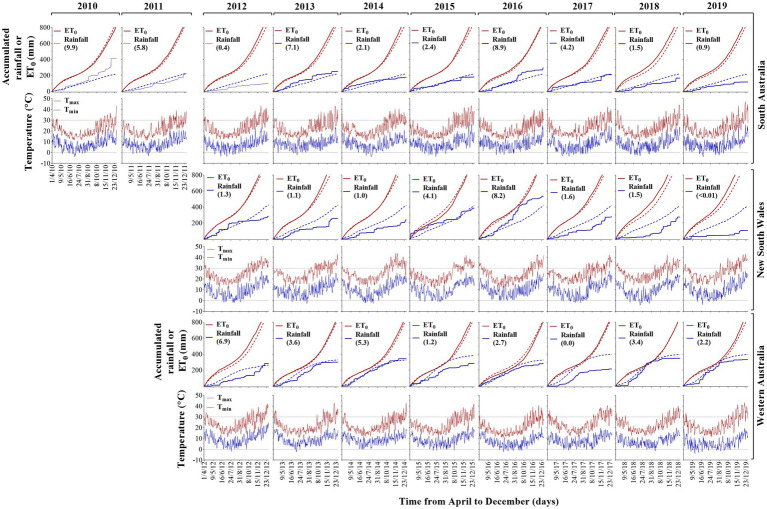
Accumulated rainfall, evapotranspiration (ET), daily maximum and minimum air temperature as a function of time from April to December between 2010 and 2019 growing seasons in SA, NSW and WA. Solid and dotted lines correspond to in-crop and long-term values [from 1960 up to the previous year for each frost expression experiments (FEE)] of accumulated ET (red color) and accumulated rainfall (blue color), respectively. Horizontal dotted black lines are temperature values at 30°C or 0°C. Values between parentheses represent rainfall deciles.

Rainfall decile across experiments were 2.4 (NSW), 4.4 (SA) and 5.9 (WA), and rainfall during the growing season was distant to the reference evapotranspiration (Figure 1). The wettest experiment was SA10, while the driest corresponded to NSW19. Averaging FEEs, the lowest temperature values were −2.5°C, −3.3°C and −1.4°C (NSW, SA and WA, respectively), representing a reduction by 7.3% (NSW), or an increase of 26.3% (SA) and 29.5% (WA) when compared to the long-term (from 1960 up to date of each experiment) lowest temperature values (Supplementary Table 2). The warmest site was SA, reaching 43.6°C (7% higher than the long-term average value), followed by NSW with 42.5 (+7.2% compared to the long-term average value), and WA with 41.7 (+2% compared to the long-term average value; Supplementary Table 2; Figure 1).

**Figure 2:**
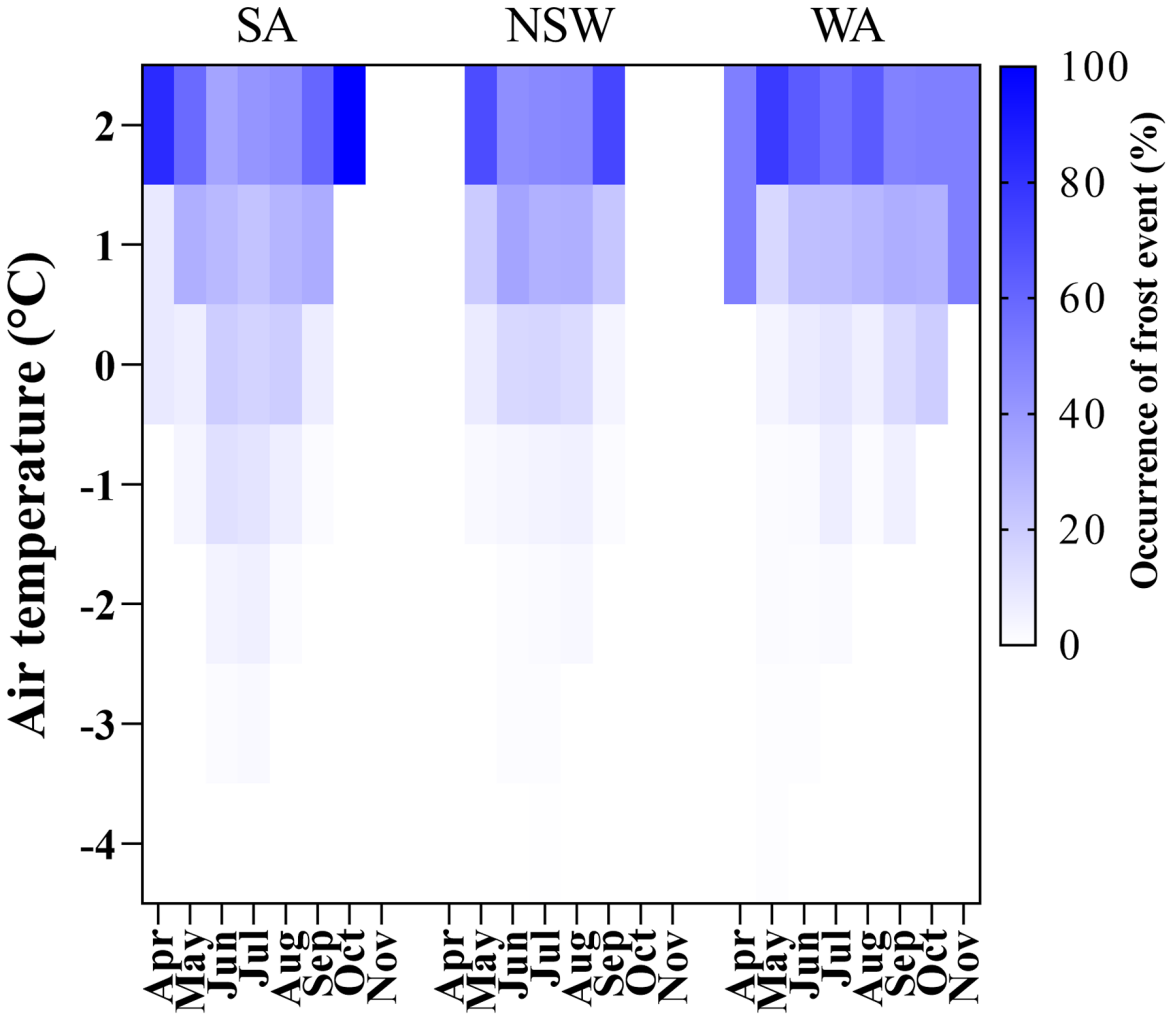
Heat map showing the occurrence of frost events expressed as a percentage of air temperature values falling from 2°C to −4°C from April to November across growing seasons in SA, NSW and WA regions.

The percentage of occurrence of particular frost events across experiments, falling from 2°C to −4°C is shown in Figure 2. Above 91, 63 and 92% of frost events occurred in the range between 2°C and 0°C in NSW, SA and WA, respectively. This fell to 5, 15 and 5% when the temperature range analyzed was between 0°C and −2°C for the same states (Figure 2). In the case of SA, with the highest frost severity, 5% of frost occurrence was recorded for the range between −2°C and −4°C.

### Frost Sterility

Initially, a separate LMM analysis was conducted for each FEE. This model was used to diagnose possible outliers, examine the validity of the normality assumption for the transformed FS and determine whether each FEE had non-zero genetic variance.

Supplementary Tables 3 and 4 (wheat and barley, respectively) present a summary of Wald tests for fixed effects and proportion variance associated with random terms in the FALMM from the fit of the diagonal variance model, after excluding the five FEEs with zero genetic variance. The largest source of variation across all FEEs was the units term with an average of 52.8 and 67.0% (wheat and barley, respectively). This source represents variation between spikes within plots within tagging events, and this highlights the innate variability of FS and the challenges to accurately assess genetic effects without exhaustive sampling of spikes within plots. The other major source of non-genetic variation was due to the tagging events, with an average of 17.0 and 9.5%. The largest source of genetic variation was due to the main effect of Genotype (9.8 and 8.3%). Higher order terms associated with Genotype contributed to the variation in FS, especially the term Block[TOS]:Genotype, however the limitations in the experimental design makes meaningful inferences about this problematic.

A series of FALMMs were then fitted to the MET data, commencing with an FA (1) model, then FA (2) and then FA (3) (Table 5). The numbers below the column labeled parameters relate to the (variance) parameters associated with the variance model for the GEI) effects and also with variance models for other random effects and the residuals. The magnitude of the GEI interaction in the data was seen through the substantial increases in REML log-likelihood between successive FALMM models. The superior model, using both likelihood ratio and AIC criteria, was the FA (3) model so this was chosen as the final model on which to base predictions and summaries of the GEI for key genotypes using FAST tools.

**Table 5:** Summary of models fitted FA (1), FA (2), and FA (3) including the number of parameters, residual maximum log-likelihood (REML.loglik) and Akaike information criterion (AIC).

Crop	Model	Parameters	REML.loglik	AIC
Wheat	FA (1)	266	−287934.7	576401.4
	FA (2)	288	−287890.2	576356.3
	FA (3)	309	−287865.8	576349.7
Barley	FA (1)	220	−158231.7	316903.3
	FA (2)	241	−158194.3	316870.5
	FA (3)	261	−158177.9	316877.8

The rotated REML loadings from the FA (3) model, together with the I-class membership and an overall measure of the variance accounted for by each of the three factors are showed in Table 6. The three factors accounted for 88.3% (wheat) and 87.8% (barley) of the GxE variance. Nineteen out of 23 (wheat) and 15 out of 22 (barley) FEEs had more than 90% explained by the three factors, and the remaining FEEs corresponded to sa10, sa17, sa18 and wa13 (wheat), while in barley these were nsw12, nsw14, nsw16, nsw18 and nsw19; sa18 and wa17. In the wheat analysis, all of the estimated (rotated) loadings for the first factor were positive. In the barley analysis, the first factor contained a single negative value and this corresponded to the nsw14 FEE, which had very low genetic variance. For both wheat and barley, the second and third factors contained both positive and negative values and therefore represent potential cross-over GEI (see next section).

**Table 6:** Rotated REML estimates of loadings from the FA (3) model for genotype by environment interactions effects. Missing values correspond to zero genetic variance or when the FEE was not sown (e.g., sa10 and sa11 in barley). There were differences in the frequency distribution of I-classes across states (see last column below and Supplementary Figure 1).

FEE	Wheat				Barley			
	Load1	Load2	Load3	I-class	Load1	Load2	Load3	I-class
nsw12	0.053	−0.368	0.013	pnp	0.086	−0.078	−0.270	pnn
nsw13	0.031	−0.502	−0.393	pnn	–	–	–	–
nsw14	–	–	–	–	−0.088	−0.896	0.110	nnp
nsw15	0.199	−0.171	0.284	pnp	0.222	−0.107	0.204	pnp
nsw16	0.166	−0.228	0.160	pnp	0.191	−0.041	−0.115	pnn
nsw17	0.202	−0.070	−0.119	pnn	0.258	−0.124	0.157	pnp
nsw18	0.209	0.066	0.250	ppp	0.125	−0.075	0.438	pnp
nsw19	0.098	0.388	−0.081	ppn	0.236	−0.122	0.008	pnp
sa10	0.323	0.155	−0.308	ppn	–	–	–	–
sa11	–	–	–	–	–	–	–	–
sa12	0.243	0.041	−0.196	ppn	0.205	−0.075	−0.236	pnn
sa13	0.197	−0.076	0.069	pnp	0.311	−0.134	−0.321	pnn
sa14	–	–	–	–	0.195	−0.104	−0.206	pnn
sa15	0.291	−0.144	−0.290	pnn	0.268	0.022	−0.161	ppn
sa16	0.254	−0.078	−0.389	pnn	0.222	−0.048	−0.460	pnn
sa17	0.231	0.301	−0.044	ppn	0.191	−0.029	0.114	pnp
sa18	0.138	0.004	0.294	ppp	0.257	−0.047	0.159	pnp
sa19	0.124	0.159	0.022	ppp	0.191	−0.107	0.131	pnp
wa12	0.347	0.223	0.035	ppp	0.203	0.041	0.265	ppp
wa13	0.199	−0.064	0.078	pnp	0.185	0.161	0.241	ppp
wa14	0.140	−0.094	0.302	pnp	0.159	0.062	0.040	ppp
wa15	0.197	−0.023	0.108	pnp	0.228	0.056	0.036	ppp
wa16	0.123	0.290	−0.064	ppn	–	–	–	–
wa17	0.240	−0.165	0.106	pnp	0.152	0.172	−0.021	ppn
wa18	0.192	−0.047	0.127	pnp	0.272	0.128	0.123	ppp
wa19	0.260	−0.147	0.228	pnp	0.274	0.104	0.055	ppp

### Genotypic Performance

To investigate frost susceptibility of the varieties we used the methodology of Smith et al (*unpublished*) which is an extension of the FAST presented in Smith and Cullis (2018). Two key FAST are the measures of overall performance (OP) and stability (root mean square deviation, RMSD) for each variety. These are global measures that summarize across all environments (FEEs in our study) in the MET data-set. In the presence of cross-over GEI, Smith et al (*unpublished*) recommend forming groups of environments with similar patterns of GEI, then computing separate OP and RMSD measures for each group. The groups, which will be called “Interaction classes” (I-classes), are formed using the fundamental parameters of the FA model, namely the (rotated) loadings. This makes use of the property that a factor with positive loadings for some environments and negative loadings for the remainder represents a contrast between the two sets of environments. I-classes are formed by splitting the loadings for each factor into positive and negative values, then cross-tabulating across factors. This classifies individual environments into I-classes, with each environment belonging to a single I-class.

In the current study, the final FALMM fitted for both the wheat and barley analyses involved FA (3) models. Thus, in each case there were potentially 2 × 2 × 2 = 8 I-classes that correspond to the splitting of the loadings for each of the three factors into positive and negative values. The I-classes are labeled with a three-character code (one for each factor), where each character is either “p” or “n” (for positive or negative loadings). For example, the I-class labeled “pnp” contains all FEEs that had positive loadings in the first and third factors and negative loadings in the second factor. Forming I-classes in this way means there is minimal cross-over GEI (hence minimal changes in genotype rankings) between FEEs within an I-class but there may be substantial cross-over GEI between FEEs in different I-classes. The I-classes to which individual FEEs belong are given in Table 6. Note that in the wheat analysis, only four of the possible eight I-classes were present, namely pnn (4), pnp (10), ppn (5), and ppp (4). For barley there were five I-classes, namely nnp (1), pnn (6), pnp (7), ppn (2), and ppp (6).

**Figure 3:**
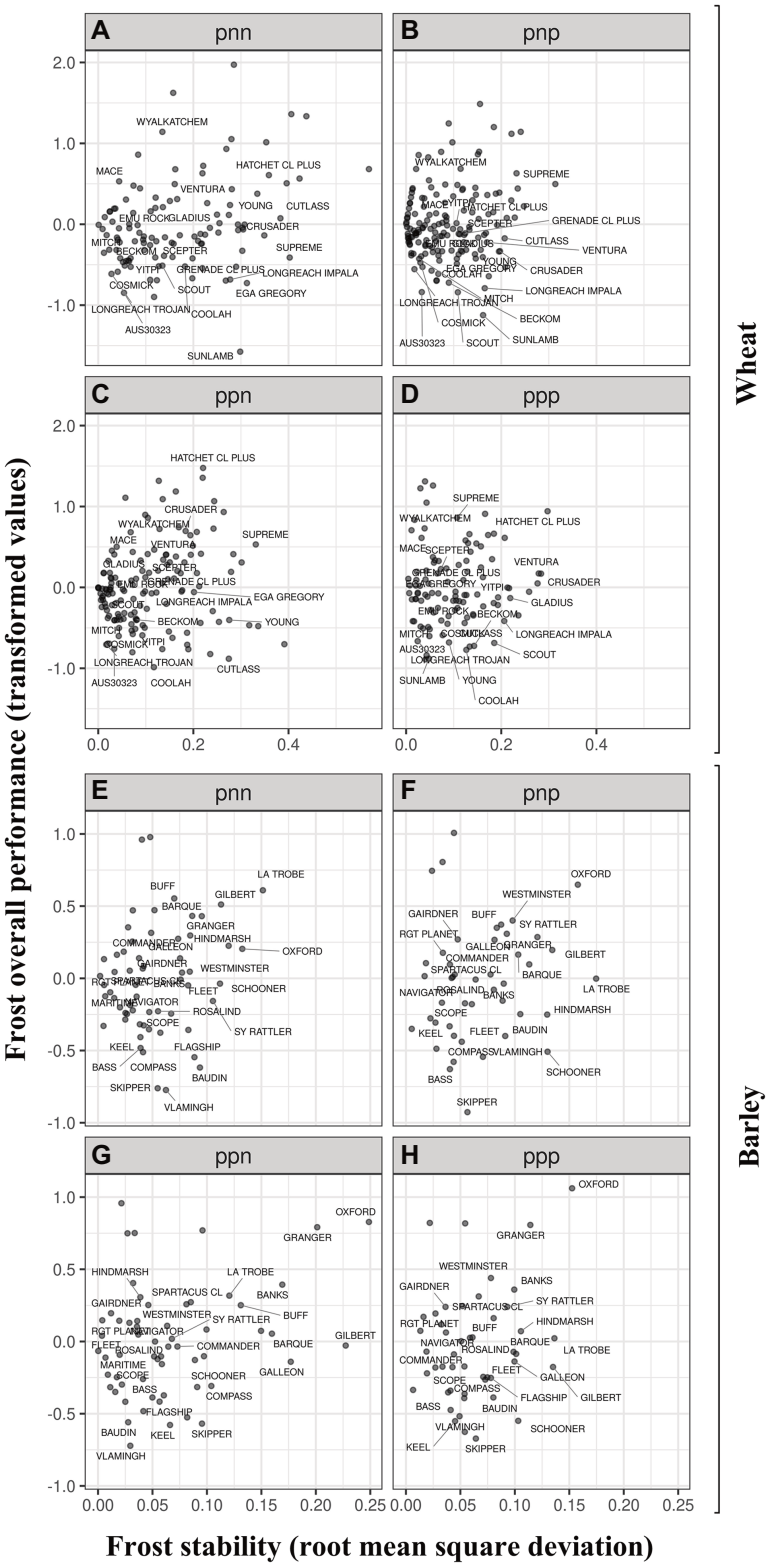
Frost overall performance (OP, frost transformed values) as a function of frost stability for wheat **(A–D)** and barley **(E–H)**. OP between different I-classes for wheat and barley are presented in panels (A-H), respectively. The I-classes are labeled with a three-character code (one for each factor), where each character is either “p” or “n” (for positive or negative loadings). For example, the I-class labeled “pnp” contains all experiments that had positive loadings in the first and third factors and negative loadings in the second factor. The FEEs within I-classes are presented in Table 6.

Within an I-class, OP is a measure of a genotype’s so-called average performance (on the scale of the transformed data) across the FEEs in that I-class. Genotypes with both low OP and low RMSD within an I-class were less frost susceptible and performed more consistently across all FEEs in the I-class. For example in wheat, differences between Cosmick and the synthetic derived line AUS30323 (the most adapted across FEEs and the least susceptible to frost damage) were associated with variation in frost susceptibility at similar environmental conditions (i.e., similar stability) and similar maturity type. Nevertheless, Cosmick has a significantly shorter plant height (and is similar to the average across FEEs) and smaller spike length than AUS30323 (Supplementary Figures 2A–D). In contrast, and when comparing other varieties from different maturity groups but with similar maturity types to one another, the variety Young was generally more poorly adapted to a wide range of environments than Emu Rock (i.e., higher stability value; both having early maturity). A similar pattern was observed when we compare other examples including the mid-late maturity type varieties Cutlass (more poorly adapted) vs. Mitch (broader adaptation; Figures 3A–D). The widely grown Wyalkatchem was one of the most susceptible varieties to frost damage across the regions being one-or-three-fold more susceptible than Mace (same maturity type – early-to-mid) or Scout (similar maturity type – mid), respectively.

**Figure 4:**
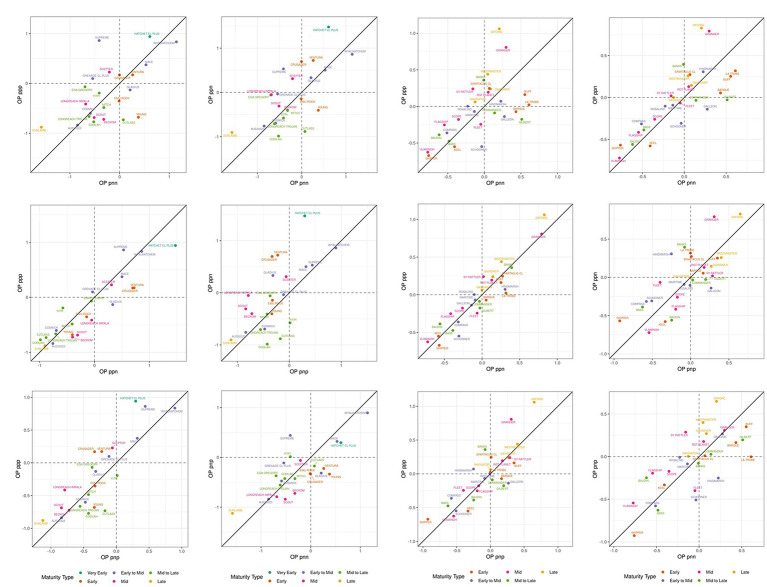
Frost overall performance (OP) between I-classes in wheat (panels A-F) and barley (panels G-L). The I-classes are labeled with a three-character code (one for each factor), where each character is either “p” or “n” (for positive or negative loadings). Colors correspond to difference in maturity type. The FEEs within I-classes are presented in Table 6 and Supplementary Figure 1.

In barley, within the same maturity type, the variety Compass was less susceptible to frost sterility (negative value of OP) than the varieties Spartacus CL or La Trobe (positive values of OP). The most adapted variety across FEEs and the least susceptible to frost damage were the varieties Vlamingh and Skipper (Figures 3E–H). Figure 4; Supplementary Figures 4 (wheat) and 5 (barley) show the differences in frost OP among all I-class combinations (ppp, ppn pnp, pnn) where varieties were grouped by their maturity type. I-class nnp for barley is not presented as there was only one FEE in this I-class and therefore this environment is considered too dissimilar to report (i.e., very low frequency of seeing this environment).

In wheat, all varieties located in the bottom left corner showed reduced frost susceptibility (Figures 4A–F). On average, the lowest OP values within I-classes were associated with varieties having mid (e.g., Coolah) or late maturity (e.g., Sunlamb). The variety Cutlass however, was an anomaly within the mid to late maturity category and reported positive OP values (i.e., higher frost susceptibility than the average) in the “pnn” I-class (Figures 4A,B,F). This was also the case for Scepter (mid maturity) within the ppp I-class (Figures 4A,C,E). The late maturing Sunlamb variety showed the most reduced frost susceptibility, while the highest frost susceptible varieties were the early and early-to-mid maturing Hatchet CL Plus and Wyalkatchem, respectively (Figure 4).

In barley, the relationship between frost susceptibility and maturity type was not consistent across I-classes and tended to operate in the opposite way when compared to wheat. On average, late and mid maturing varieties Oxford and Granger showed the highest values of OP in most of the I-class combinations (Figures 4G–L). The early maturing variety Skipper and mid maturing variety Vlamingh achieved the best OP scores, irrespective of the I-class.

### Plant Height and Spike Length

A summary of the Wald test for fixed effects for plant height and spike length are presented in Table 7. Most terms are highly significant with the exception of Expt:SOD for spike length (Pwald = 0.109; wheat) and Expt:SOD for plant height (Pwald = 0.992; barley).

**Table 7:** Summary of Wald tests for fixed effects for plant height and spike length in wheat and barley. Most terms are highly significant with the exception of Expt:SOD for spike length in wheat and plant height in barley (shown in bold).

Term	Wheat						Barley					
	Plant Height			Spike Length			Plant Height			Spike Length		
	*df*	Wald	Pwald	*df*	Wald	Pwald	*df*	Wald	Pwald	*df*	Wald	Pwald
(Intercept)	1	9,538	0.000	1	9,515	0.000	1	9,592	0.000	1	3152.0	0.000
Expt	21	367.7	0.000	16	335.4	0.000	23	521.9	0.000	18	463.1	0.000
Expt:Block[TOS]	88	882.6	0.000	73	342.5	0.000	76	604.7	0.000	66	182.0	0.000
Expt:SOD	8	136.6	0.000	8	1.6	**0.992**	7	11.7	**0.109**	5	341.3	0.000
Expt:Block[TOS]:SOD	22	68.9	0.000	22	43.8	0.004	0			2	5.1	0.079

In addition, Table 8 shows a summary of the proportion of variance associated with each of the random terms fitted in the simplified LMM for plant height and spike length. Note that plant height was assessed at the plot level for each tag event and FEE, hence variation between spikes within plots for each FEE and tag event cannot be estimated. In wheat, the major sources of variation for plant height were associated with Genotype, TagEvent, Plot and Plot:TagEvent, while the major sources of variation for spike length were associated with Genotype and Units, the latter representing spikes within plots within tag events. In barley, the major sources of variation for plant height were associated with Genotype, TagEvent, Plot and Units. The major sources of variation for spike length were associated with Genotype and Units.

**Table 8:** Summary of the variance associated with random terms for plant height and spike length. Values are expressed as a proportion of total variance (the final row of the table), and those which are zero are less than 0.0001. Note the term associated with spike variation within tag events for each FEE is the units for plant height. Numbers in bold show the major source of variation.

Terms	Wheat		Barley	
	Plant height	Spike length	Plant height	Spike length
Genotype	**0.221**	**0.302**	**0.254**	**0.436**
Expt:Genotype	0.000	0.050	0.051	0.011
Expt:TagEvent	**0.344**	0.049	**0.160**	0.008
Expt:Genotype:SOD	0.054	0.001	0.000	0.025
Expt:Genotype:TagEvent	0.000	0.007	0.000	0.005
Expt:Block[TOS]:Genotype	0.000	0.000	0.042	0.000
Expt:Block[TOS]:TagEvent	0.036	0.020	0.008	0.017
Expt:Block[TOS]:Genotype:SOD	0.043	0.011	0.000	0.020
Expt:Block[TOS]:Genotype:TagEvent	0.042	0.018	0.092	0.008
Expt:Block[TOS]:SBlock	0.013	0.006	0.028	0.009
Expt:Block[TOS]:SBlock:TagEvent	0.000	0.005	0.003	0.000
Expt:Block[TOS]:SBlock:Plot	**0.093**	0.045	**0.202**	0.031
Expt:Block[TOS]:SBlock:Plot:TagEvent		0.029		0.053
Expt:TaggerFix	0.067	0.002	0.036	0.019
Expt:CounterFix	0.000	0.078	0.003	0.010
Units	0.088	**0.377**	**0.121**	**0.346**
Total variance	81.7	190.3	59.2	213

Moreover, wheat varieties shown in the bottom left corner in Figure 3 within each I-class tended to have higher spike length for mid-to-late or late-maturing varieties (above mean; see Supplementary Figure 2). From “Very early” to “Early to mid” maturity type, varieties with reduced frost susceptibility (negative OP values) were shorter than the average, except AUS30323 which was the tallest across the I-classes (Supplementary Figures 2A–D). In barley, there was no clear pattern, but the late maturing varieties had a longer spike length and reduced frost susceptibility compared to the early maturing varieties (Supplementary Figures 2M–P).

## Discussion

In this study, we have shown for the first-time genotypic differences to frost damage in barley and extended the wheat data-set by 3 years from what was reported in Cocks et al. (2019). In addition, this study also represents the first application of the new I-class concept, which allows for use of the FAST tools of Smith and Cullis (2018), but within so-called interaction classes, in which there is minimal GEI. Through phenotyping a vast number of wheat and barley spikes (in excess of 557,000) and recording accurate empirical measurements across Australian prone-frost regions, this has resulted in a substantial number of experimental units (10,317 and 5,563 in wheat and barley) across states, sites, multiple years and times of sowing being generated. Combined, this has culminated in 126 and 108 different growing conditions in wheat and barley, respectively. To mitigate against frost risk, a well-known approach carried out by wheat and barley growers is to adjust sowing time and to also carefully consider the variety grown (i.e., maturity type), or both. These two considerations alone can, at times, lead to the plant successfully avoiding frost damage during the critical period of development (Levitt, 1980; Sadras and Monzon, 2006; Zheng et al., 2015; Crimp et al., 2016). However, on the flip-side, and particularly in Australia; when the TOS is delayed this decreases the yield potential due to the crop more likely being exposed to heat and drought stress as the warmer summer months approach during grain fill (Chenu et al., 2011, 2013). A wide range of susceptibility to frost damage (frost overall performance) was determined in this research as a function of frost stability, mainly associated with genotypic differences due to maturity type, coinciding or avoiding the high susceptibility to frost damage around heading and anthesis in barley and wheat, respectively (i.e., critical period for fertile floret and grain number determination, Fischer, 1985; Arisnabarreta and Miralles, 2008).

The number of grains per spike is attributable to the dynamics of floret initiation and degeneration to produce fertile florets at flowering (Kirby, 1988). In this study it was shown that increasing the occurrence of frost events and severity during the critical period around flowering affected the mortality of floret primordia development to become fertile florets or grain. In wheat, we based our measurements by mainly matching tagged plots at flowering to later determine (DC83-85) frost sterility. In doing so we only considered the first two grain positions closest to the rachis (G1 and G2) within each spikelet.

However, a recent detailed study carried out in the same nursery in SA (that included individual grain mapping) showed that when frost was ‘avoided’, spike fertility increased even in the intermediate positions (G3 and G4) of the spikelets, when compared to controls. This was because the labile floret primordia (F3 and F4 positions) were not aborted, and so could contribute to an increased number of grains per spike and in turn, grain yield (Ferrante et al., 2019). This is in line with previous research where differences in resource availability, signals and genetic variation have been reported for wheat (Ferrante et al., 2010, 2013, 2020; Ochagavía et al., 2021) and barley (Arisnabarreta and Miralles, 2010).

The novel detailed statistical framework used in the analysis of this research, which has been updated from Smith and Cullis (2018), has allowed meaningful measures of frost sterility using the FAST tools, such as Overall Performance (OP) and stability, which are formed within groups of environments which exhibit minimal cross-over GEI. This in turn has enabled us to compare among varieties those having similar frost susceptibility but a different response to a change in environment or *vice versa*. Such an approach has enabled the identification (Figure 3) of well-adapted varieties and/or lines as the most promising for growers or for breeding programs to use as parental germplasm in order to enhance genetic gain against this economically devastating trait. However, it should also be noted that the FEE considered in the MET analysis corresponds to previous frost events and all environmental interactions therein, and therefore should not be generalized as a prediction score for future frost damage, because the risk of selection errors can be unacceptably high as previously mentioned by Smith et al. (2015).

Differences in plant height and spike length among varieties (semi-dwarf) or advanced breeding lines were highly significant, and the magnitude of these differences can largely be explained by the experiment or experiment × TOS interaction, because of the sensitivity of phasic development to major environmental factors (i.e., development was progressively accelerated as sowing was delayed by temperature and photoperiod effect per se; Slafer and Rawson, 1994).

When OP was plotted as a function of plant height and spike length, wheat varieties (excluding AUS30323) with high susceptibility to frost damage (OP > 0) tended to be shorter with a reduced number of fertile spikelets per spike. However, in barley this pattern was not consistent for plant height (Supplementary Figure 2). Moreover, in wheat, the early maturing varieties had higher values of OP (values > 0) compared to the late maturing varieties (OP < 0), but in barley this pattern was generally reversed (Supplementary Figures 3–5), because (i) the dynamics of tillering might be more relevant than in wheat as a determinant of the response to resources (Ferrante et al., 2008) (ii) morphological differences (Single, 1991), or (iii) the variation in reproductive frost tolerance between these species (Reinheimer et al., 2004).

This research has benchmarked and ranked wheat and barley varieties (and advanced breeding lines) against frost susceptibility based on empirical manual observations using low-technology to improve pre-season planning for frost. However, what would be of most use to grain growers and plant breeders when assessing frost damage is a fast and preferably non-destructive selection tool that is also low-cost. Developing a technology that has all these features would ultimately improve the flexibility and speed in providing the most appropriate decision making-strategy under frost-prone landscapes and in the process, significantly improve the economic and genetic return to growers and breeding programs, respectively.

Finally, heat and drought stress were not quantified in this research, but these abiotic stress constraints also play a crucial role during the grain filling stage. As such, heat and drought can also have a profound co-effect on final grain set and therefore grain yield. Future investment should consider combining these traits in what would no doubt be a complex, high risk program – but one in which the outputs would benefit Australian grain growers in the medium to long term.

## Conclusion

Elucidating and implementing genotypic gain of frost tolerance in wheat and barley varieties remains a constant challenge for pre-breeding research and breeding programs alike. However, the results presented is this study now allow breeding programs (particularly) to utilize this information as a foundation to develop the most suitable varieties with improved susceptibility and/or avoidance to spring radiation frost and provide an increased understanding to mitigate frost damage under frost-prone regions in Australia.

## Data Availability Statement

The original contributions presented in the study are included in the article/supplementary material, further inquiries can be directed to the corresponding author.

## Author Contributions

AF carried out selected experiments in South Australia, supervised the selected NSW and WA trials, and wrote the first draft of the manuscript. AF, BC, and AS analyzed and interpreted the data. AF, BC, AS, and JA contributed to the writing, review and editing of the manuscript. All authors read and agreed to the published version of the manuscript.

## Conflict of Interest

The authors declare that the research was conducted in the absence of any commercial or financial relationships that could be construed as a potential conflict of interest.

## Publisher’s Note

All claims expressed in this article are solely those of the authors and do not necessarily represent those of their affiliated organizations, or those of the publisher, the editors and the reviewers. Any product that may be evaluated in this article, or claim that may be made by its manufacturer, is not guaranteed or endorsed by the publisher.

## Footnotes

^1^ https://www.nvtonline.com.au/frost/

^2^ https://www.katmandoo.org.au

^3^ https://www.longpaddock.qld.gov.au/silo/index.html
