# Supplementary material for: A Multi-Environment Trial Analysis of Frost Susceptibility in Wheat and Barley Under Australian Frost-Prone Field Conditions
Source: Front Plant Sci. 2021 Aug 19;12:722637. doi: 10.3389/fpls.2021.722637 (PMC8417324; doi:10.3389/fpls.2021.722637)
Supplement: Supplementary file 1 [file Data_Sheet_1.docx]

**Supplementary Table 1*.*** List of screened wheat and barley varieties and breeding lines across FEEs.

**Supplementary Table 2*.*** In-crop temperature values, accumulated rainfall, global radiation, photo-thermal quotient (PTQ), and rainfall decile for each frost expression experiment (FEE). Long-term values from 1960 up to the previous growing season for each FEE are in bold.

| FEE | Growing | Temperature (°C) | | | | | A rainfall | G radiation | PTQ | Decile |
| --- | --- | --- | --- | --- | --- | --- | --- | --- | --- | --- |
|  | season | Max | Highest | Min | Lowest | Mean | (mm) | (MJ m^-2^ d^-1^) | (MJ m^-2^ Cd^-1^) |  |
| nsw12 | In-crop | 25.0 | 40.9 | 9.1 | -2.1 | 17.1 | 284.5 | 18.8 | 1.10 | 1.3 |
|  | **Long term** | **24.3** | **39.5** | **9.4** | **-2.7** | **16.9** | **427.9** | **17.8** | **1.06** |  |
| nsw13 | In-crop | 25.8 | 43.1 | 9.4 | -2.9 | 17.6 | 257.0 | 19.1 | 1.08 | 1.1 |
|  | **Long term** | **24.3** | **39.6** | **9.4** | **-2.7** | **16.9** | **422.1** | **17.9** | **1.06** |  |
| nsw14 | In-crop | 25.7 | 44.0 | 10.4 | -2.7 | 18.0 | 240.4 | 18.4 | 1.02 | 1.0 |
|  | **Long term** | **24.4** | **39.6** | **9.4** | **-2.7** | **16.9** | **418.8** | **17.9** | **1.06** |  |
| nsw15 | In-crop | 24.6 | 42.0 | 10.3 | -1.6 | 17.4 | 386.6 | 17.4 | 1.00 | 4.1 |
|  | **Long term** | **24.4** | **39.6** | **9.4** | **-2.7** | **16.9** | **418.8** | **17.9** | **1.06** |  |
| nsw16 | In-crop | 24.4 | 42.5 | 10.4 | -0.1 | 17.4 | 534.4 | 16.4 | 0.94 | 8.2 |
|  | **Long term** | **24.4** | **39.7** | **9.5** | **-2.6** | **16.9** | **420.3** | **17.8** | **1.05** |  |
| nsw17 | In-crop | 25.3 | 42.4 | 9.5 | -2.1 | 17.4 | 278.2 | 17.3 | 0.99 | 1.6 |
|  | **Long term** | **24.4** | **39.7** | **9.5** | **-2.6** | **16.9** | **420.3** | **17.8** | **1.05** |  |
| nsw18 | In-crop | 26.1 | 42.4 | 10.0 | -4.3 | 18.1 | 274.6 | 17.5 | 0.97 | 1.5 |
|  | **Long term** | **24.4** | **39.8** | **9.5** | **-2.6** | **16.9** | **417.8** | **17.8** | **1.05** |  |
| nsw19 | In-crop | 26.7 | 42.9 | 10.3 | -3.9 | 18.5 | 106.3 | 17.7 | 0.95 | 0.1 |
|  | **Long term** | **24.4** | **39.8** | **9.5** | **-2.6** | **16.9** | **415.4** | **17.8** | **1.05** |  |
| sa10 | In-crop | 21.0 | 43.6 | 7.7 | -3.9 | 14.3 | 414.4 | 15.5 | 1.08 | 9.9 |
|  | **Long term** | **21.5** | **40.6** | **7.8** | **-2.5** | **14.7** | **216.4** | **15.9** | **1.09** |  |
| sa11 | In-crop | 22.1 | 39.1 | 8.1 | -3.3 | 15.1 | 223.8 | 16.2 | 1.07 | 5.8 |
|  | **Long term** | **21.5** | **40.6** | **7.8** | **-2.5** | **14.7** | **220.3** | **15.9** | **1.09** |  |
| sa12 | In-crop | 22.5 | 43.6 | 7.3 | -3.6 | 14.9 | 97.9 | 16.9 | 1.13 | 0.4 |
|  | **Long term** | **21.5** | **40.6** | **7.8** | **-2.5** | **14.7** | **220.4** | **15.9** | **1.08** |  |
| sa13 | In-crop | 22.7 | 43.0 | 8.1 | -2.3 | 15.4 | 250.1 | 16.3 | 1.06 | 7.1 |
|  | **Long term** | **21.6** | **40.7** | **7.8** | **-2.6** | **14.7** | **218.6** | **15.9** | **1.09** |  |
| sa15 | In-crop | 22.7 | 43.7 | 8.2 | -3.9 | 15.5 | 179.9 | 16.0 | 1.03 | 2.4 |
|  | **Long term** | **21.6** | **40.7** | **7.8** | **-2.6** | **14.7** | **217.9** | **15.9** | **1.09** |  |
| sa16 | In-crop | 21.9 | 40.6 | 8.0 | -2.1 | 14.9 | 294.3 | 15.4 | 1.03 | 8.9 |
|  | **Long term** | **21.6** | **40.8** | **7.8** | **-2.6** | **14.7** | **218.6** | **15.9** | **1.08** |  |
| sa17 | In-crop | 22.7 | 42.6 | 7.7 | -3.3 | 15.2 | 209.2 | 15.5 | 1.02 | 4.2 |
|  | **Long term** | **21.6** | **40.8** | **7.8** | **-2.6** | **14.7** | **218.6** | **15.9** | **1.08** |  |
| sa18 | In-crop | 22.9 | 44.0 | 7.9 | -3.0 | 15.4 | 165.9 | 15.4 | 1.00 | 1.5 |
|  | **Long term** | **21.6** | **40.8** | **7.8** | **-2.6** | **14.7** | **218.4** | **15.9** | **1.08** |  |
| sa19 | In-crop | 23.0 | 47.3 | 7.7 | -3.9 | 15.3 | 117.4 | 16.1 | 1.05 | 0.9 |
|  | **Long term** | **21.7** | **40.8** | **7.8** | **-2.6** | **14.7** | **217.5** | **15.9** | **1.08** |  |
| wa12 | In-crop | 24.2 | 42.4 | 9.7 | -2.1 | 16.9 | 283.7 | 18.2 | 1.07 | 6.9 |
|  | **Long term** | **22.5** | **40.8** | **9.3** | **-1.1** | **15.9** | **263.5** | **17.6** | **1.11** |  |
| wa13 | In-crop | 24.3 | 43.1 | 10.1 | -1.4 | 17.2 | 209.1 | 17.6 | 1.03 | 2.4 |
|  | **Long term** | **22.5** | **40.9** | **9.3** | **-1.1** | **15.9** | **262.9** | **17.6** | **1.11** |  |
| wa14 | In-crop | 24.3 | 42.4 | 10.3 | -1.2 | 17.3 | 246.6 | 17.7 | 1.03 | 4.1 |
|  | **Long term** | **22.6** | **40.9** | **9.3** | **-1.1** | **15.9** | **262.6** | **17.6** | **1.10** |  |
| wa15 | In-crop | 23.1 | 42.2 | 10.2 | -1.4 | 16.6 | 281.6 | 16.9 | 1.01 | 6.8 |
|  | **Long term** | **22.6** | **40.9** | **9.3** | **-1.1** | **15.9** | **262.6** | **17.6** | **1.10** |  |
| wa16 | In-crop | 22.1 | 41.7 | 8.4 | -1.7 | 15.2 | 263.4 | 17.1 | 1.12 | 5.2 |
|  | **Long term** | **22.6** | **40.9** | **9.3** | **-1.1** | **15.9** | **262.9** | **17.6** | **1.10** |  |
| wa17 | In-crop | 22.8 | 38.4 | 9.8 | -0.4 | 16.3 | 289.6 | 16.4 | 1.00 | 7.3 |
|  | **Long term** | **22.6** | **40.9** | **9.3** | **-1.1** | **15.9** | **262.9** | **17.6** | **1.10** |  |
| wa18 | In-crop | 22.6 | 40.1 | 9.5 | -1.6 | 16.0 | 309.1 | 16.1 | 1.00 | 8.0 |
|  | **Long term** | **22.6** | **40.9** | **9.3** | **-1.1** | **16.0** | **263.4** | **17.5** | **1.10** |  |
| wa19 | In-crop | 23.7 | 43.1 | 9.8 | -1.4 | 16.7 | 280.9 | 16.9 | 1.01 | 6.5 |
|  | **Long term** | **22.6** | **40.9** | **9.3** | **-1.1** | **16.0** | **264.2** | **17.5** | **1.10** |  |

**Supplementary Table 3*.*** Wheat. Summary of Wald tests for fixed effects and proportion variance associated with random terms in the FALMM. Terms above the line are fixed and values in the table for these terms are p-values for the Wald tests for each experiment. Values which are zero either represent p-values less than 1 e^-3^ or are zero for fixed and random terms, respectively.

**Supplementary Table 4*.*** Barley. Summary of Wald tests for fixed effects and proportion variance associated with random terms in the FALMM. Terms above the line are fixed and values in the table for these terms are p-values for the Wald tests for each experiment. Values which are zero either represent p-values less than 1 e^-3^ or are zero for fixed and random terms, respectively.


**Supplementary Figure 1.** Schematic representation highlighting the new I-class concept undertaken in this study compared to the FAST-MET analysis previously reported by Cocks et al., (2019). Bolded frost expression experiments (FEE) represent additional datasets (wheat and barley) not previously analysed in the FAST-MET analysis but which were analysed through the I-class concept. Those FEEs highlighted in red were not included in the analysis (see Section 2.3). Interaction classes (I-classes) are labelled with a three-character code (one for each factor), where each character is either “p” or “n” (for positive or negative loadings). ^(*)^ For nsw14, I-class nnp has not been presented as there was only one FEE in this I-class. For further information on the number of FEEs obtained in each I-class, please refer to Section 3.3.

**Supplementary Figure 2.** Frost overall performance (OP, frost transformed values) as a function of plant height and spike length in wheat (panels a-d and e-h, upper and bottom panels, respectively). Colours correspond to difference in maturity type. The I-classes are labelled with a three-character code (one for each factor). The FEEs within I-classes are presented in Table 6. The grey circles labelled NR = not reported. The correlation values are shown within each panel.

**Supplementary Figure 2 (cont.).** Frost overall performance (OP, frost transformed values) as a function of plant height and spike length in barley (panels i-l and m-p, upper and bottom panels, respectively). Colours correspond to difference in maturity type. The I-classes are labelled with a three-character code (one for each factor). The FEEs within I-classes are presented in Table 6. The grey circles labelled NR = not reported. The correlation values are shown within each panel.

**Supplementary Figure 3.** Heat map. Frost overall performance (OP, frost transformed values) grouped by different I-classes and maturity type in wheat and barley. The I-class descriptions are as reported in Figure 3.


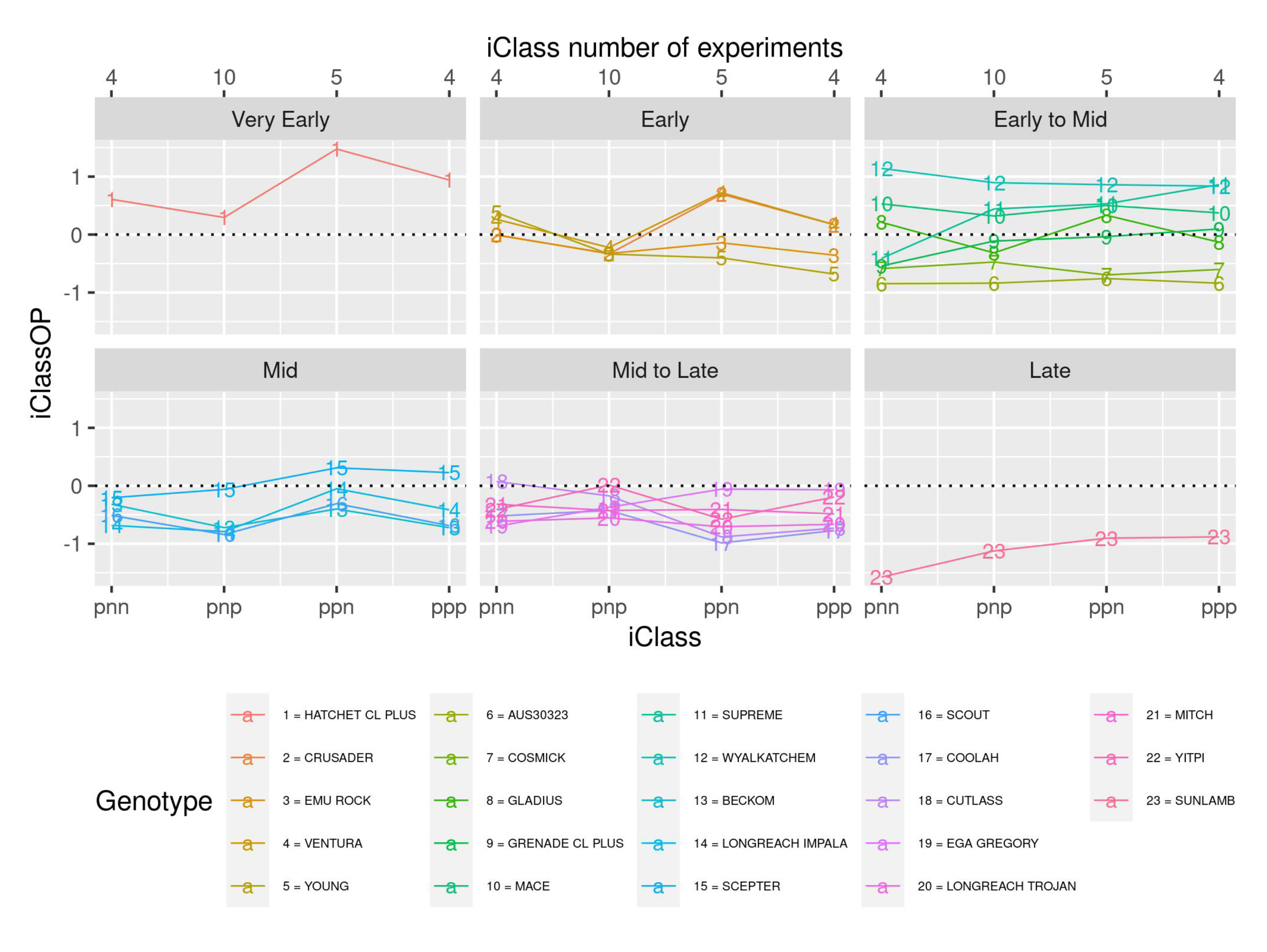


**Supplementary Figure 4.** Relationship between Interaction Class Frost Overall Performance (I-ClassOP) and different environment groups with similar patterns of GEI (i.e. pnn, pnp, ppn and ppp) in wheat. Panel sorted chronologically from Very Early to Late maturity type. The number at the top of the panels represent the total number of experiments (FEEs) within each I-class.


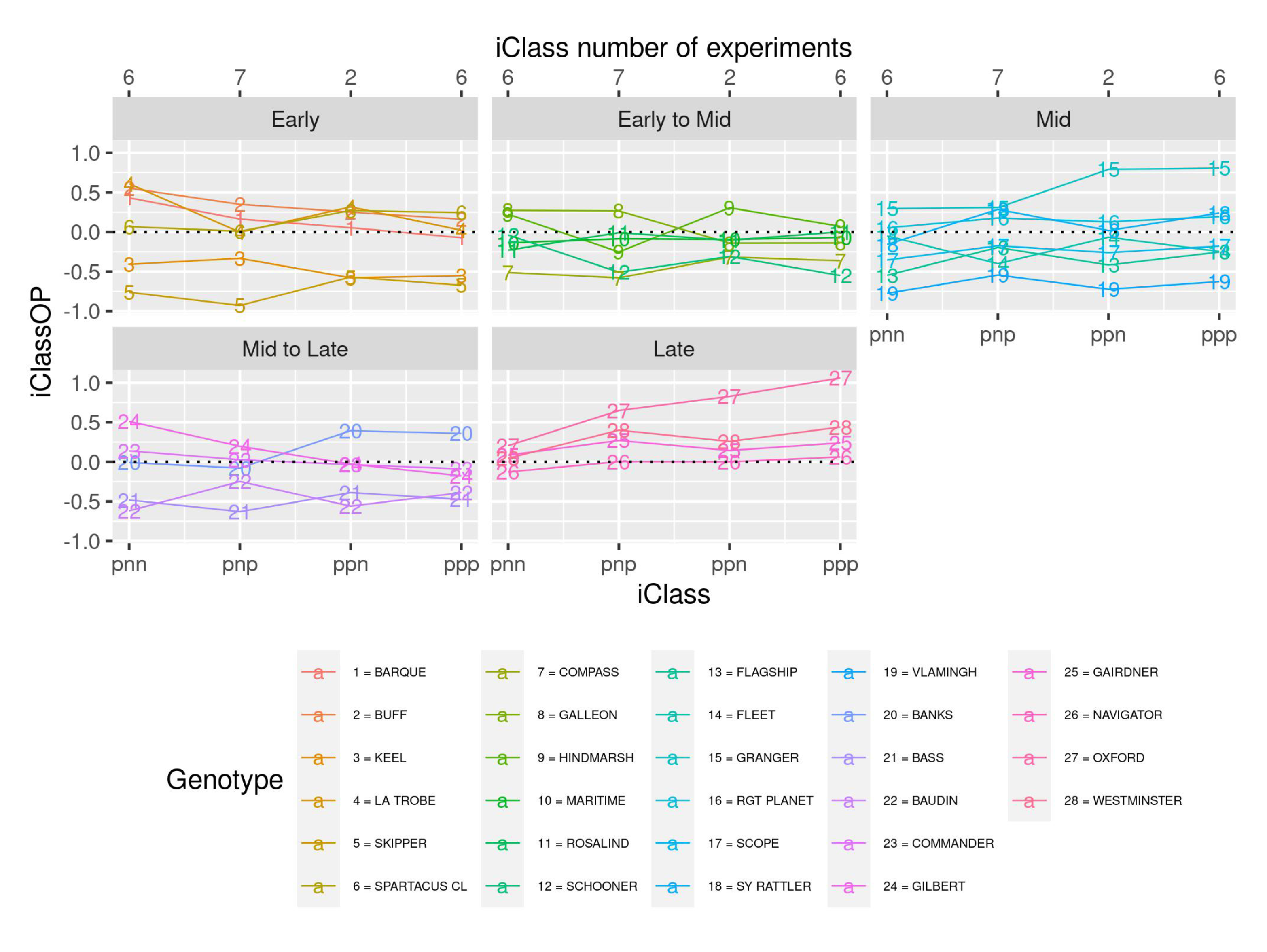


**Supplementary Figure 5.** Relationship between Interaction Class Frost Overall Performance (I-ClassOP) and different environment groups with similar patterns of GEI (i.e. pnn, pnp, ppn and ppp) in barley. Panel sorted chronologically from Very Early to Late maturity type. The number at the top of the panels represent the total number of experiments (FEEs) within each I-class.
